# A Large Silicon Pool in Small Picophytoplankton

**DOI:** 10.3389/fmicb.2022.918120

**Published:** 2022-06-09

**Authors:** Yuqiu Wei, Jun Sun

**Affiliations:** ^1^Key Laboratory of Sustainable Development of Marine Fisheries, Ministry of Agriculture and Rural Affairs, Yellow Sea Fisheries Research Institute, Chinese Academy of Fishery Sciences, Qingdao, China; ^2^Laboratory for Marine Fisheries Science and Food Production Processes, Pilot National Laboratory for Marine Science and Technology, Qingdao, China; ^3^Research Center for Indian Ocean Ecosystem, Tianjin University of Science and Technology, Tianjin, China; ^4^State Key Laboratory of Biogeology and Environmental Geology, China University of Geosciences, Wuhan, China

**Keywords:** biogeochemical cycles, silicon cycle, carbon cycle, Si accumulation, picophytoplankton

## Abstract

Marine picophytoplankton (<2 μm) play a key role in supporting food web and energy flow in the ocean, and are major contributors to the global marine carbon (C) cycle. In recent years, picophytoplankton have been found to have significant silica (Si) accumulation, a finding which provides a new sight into the interaction of marine C and Si cycles and questions the overwhelming role of large diatoms (>2 μm) in the Si cycle. As picophytoplankton have high cell abundance and wide distribution in the open ocean, exploring their influences on the C and Si cycles as well as other element cycles are becoming new scientific hotspots. However, there are still few studies on the physiology and ecology of picophytoplankton, especially their potential roles in the biogeochemical Si cycle at present. Thus, it is necessary to accurately evaluate and quantify the contributions of picophytoplankton to the C and Si cycles, and to further understand their C and Si sinking mechanisms. In this review, we expect to have a novel understanding of picophytoplankton Si pool and regulation mechanism by conducting targeted studies on these scientific issues. This also provides a premise foundation and theoretical framework for further study of the role of small cells in the global ocean Si cycle and the coupling of C and Si cycles.

## Introduction

**Figure 1:**
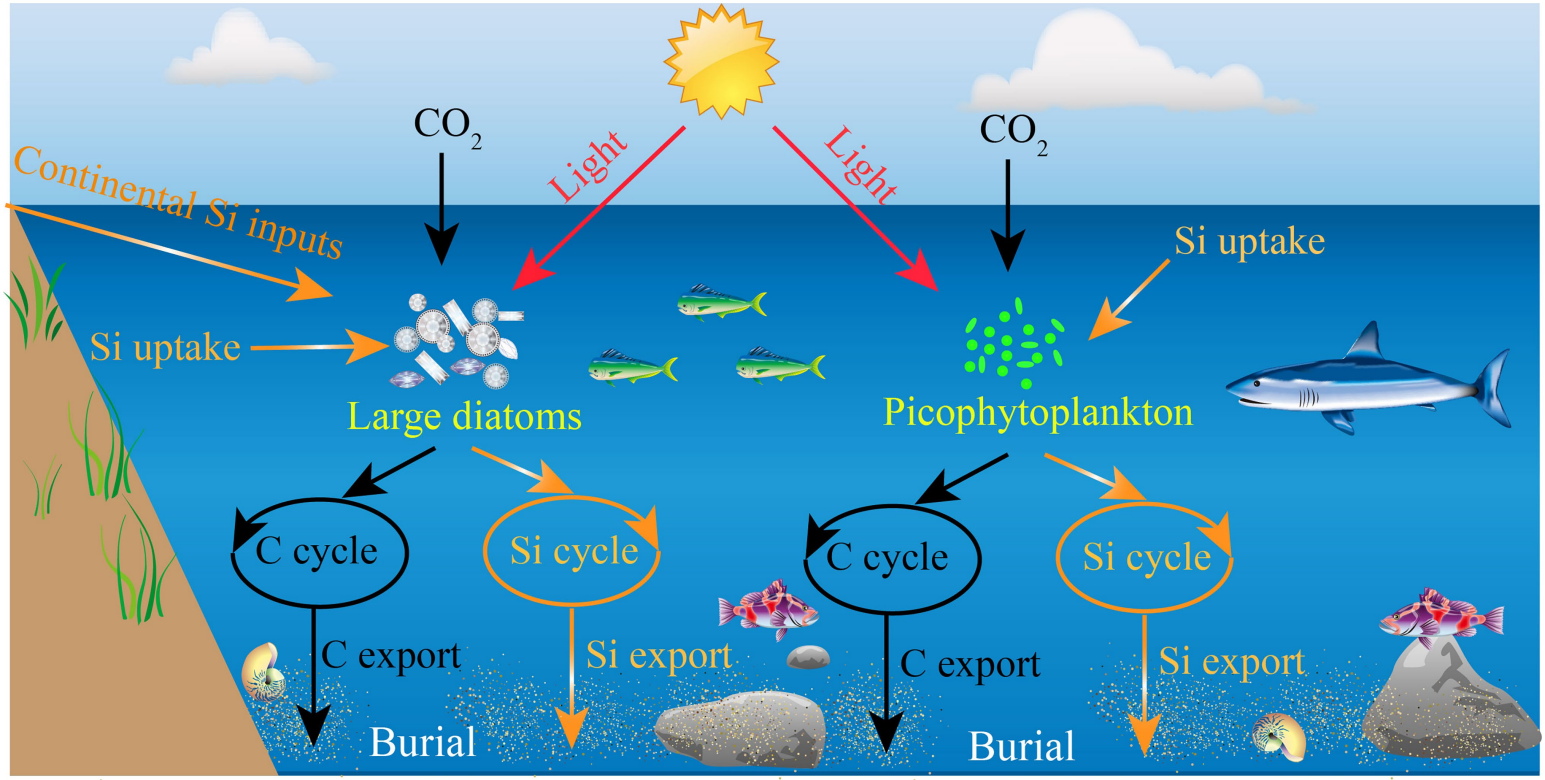
Schematic view of the significance of large diatoms and picophytoplankton in the marine C and Si cycles.

Among phytoplankton the large diatoms (>2 μm), a dominant group that are generally prevalent in coastal eutrophic ecosystems (Cermeño et al., 2005; Carstensen et al., 2015), contribute significantly to the biogenic silica (bSi) production and global primary production (Nelson et al., 1995; Brzezinski et al., 1998; Chai et al., 2007; Krause et al., 2011). These photosynthetic protists can absorb dissolved silicic acid to form their siliceous cell walls (Hecky et al., 1973; Bidle and Azam, 1999), and produce approximately 100–140 Tmol Si year^−1^ for the global ocean (Nelson et al., 1995). In addition, these large diatoms are one of the major primary producers in the ocean and are able to contribute up to ~25% of global carbon (C) fixation, although they are not dominant in oligotrophic oceans (Armbrust et al., 2004; Kale and Karthick, 2015). Aspects of their life history, notably their siliceous cell walls and high sinking rates, make them also important to the export of C and Si to depth, owing to density-driven particle sedimentation (Kemp et al., 2006; Ragueneau et al., 2006; Tréguer et al., 2018). As such, the marine Si cycle is intimately tied to the C cycle through the biotic action of large diatoms, such as growth, reproduction, and metabolism (Huang and Daboussi, 2017). Collectively, large diatoms are thought to be the primary organisms responsible for the export of Si to the ocean interior and one important group of primary producers in the global marine ecosystems (Armbrust et al., 2004; Ragueneau et al., 2006; Tréguer et al., 2018). In some cases, the presence of other siliceous organisms (e.g., Silicoflagellates and Rhizarians) would likely reduce the proportional importance of large diatoms to total bSi standing stocks (Biard et al., 2018; Hendry et al., 2018). However, a fundamental knowledge about the role of biota in marine Si cycle is that large diatoms overwhelmingly dominate the bSi standing stocks in the open ocean (Figure 1).

In recent years, regional bSi measurements have revealed that large diatoms have a disproportionately high contribution to bSi standing stock and Si export relative to their contribution to total phytoplankton biomass (Brzezinski et al., 2011; Krause et al., 2017), particularly in the mid-ocean oligotrophic gyres where diatom biomass and Si production rates are among the lowest in the ocean. For example, similar studies carried out in the equatorial Pacific (110–140°W) have led to a revised estimate of diatom bSi standing stock, as only 2%–31% of bSi pool was associated with living large diatoms (Krause et al., 2010). The implication is that the marine bSi pool may be not entirely originate from the large diatoms, and may be associated with a previously unexplored source of Si in the ocean. Surprisingly, Baines et al. (2012) have revealed that the widely distributed marine picocyanobacterium *Synechococcus* can accumulate substantial amounts of Si, a finding which may interpret the disproportionately high contributions of large diatoms to primary production and organic matter export. Also, Leblanc et al. (2018a) have demonstrated that some small diatoms (e.g., *Minidiscus*), which belong to the picoplanktonic size-class, may play a role in marine systems. However, as a result of their small size and slow sinking rate, these picophytoplankton (e.g., *Synechococcus* and small diatoms) have long been considered to represent a negligible fraction of the C and Si transport from the surface into the deep ocean (Buesseler, 1998). Until recently, some studies in oligotrophic areas have suggested that picophytoplankton are also important in transporting organic matter to the depth (Richardson and Jackson, 2007). It is well known that picophytoplankton are abundant in many oligotrophic oceans (Buitenhuis et al., 2012; Flombaum et al., 2013; Visintini et al., 2021), hence the role for picophytoplankton in the Si cycle would be more prominent in oligotrophic environments where large diatoms are in low biomass (Figure 1).

A series of theoretical arguments based on laboratory experiments and field studies have provided evidence for a role for small picophytoplankton in the Si cycle, showing that these ubiquitous small cells accumulate significant amounts of Si, exert a previously unrecognized influence on the oceanic Si cycle, and may further enhance Si export to depth (Baines et al., 2012; Tang et al., 2014; Krause et al., 2017; Leblanc et al., 2018b; Wei et al., 2021a,b). For instance, size-fractionated bSi measurements by Baines et al. (2012) and Krause et al. (2017) in the Sargasso Sea showed that the bSi standing stocks within the <3 μm size fraction averaged 16–20 and 14% of the total, respectively. Leblanc et al. (2018b) revealed a non-negligible contribution of the pico-sized fraction to bSi standing stocks (11%–26%) in the tropical South Pacific. Our earlier works in the oligotrophic eastern Indian Ocean and western Pacific Ocean have also shown that the pico-sized fraction was 49%–65% of the total bSi pool and 43% of the total bSi production rates, respectively (Wei et al., 2021a,b). Accordingly, we speculate that this previously unexplored Si source of marine picophytoplankton may be important to Si cycling in the open ocean, although the genetic and metabolic mechanisms of Si accumulation by *Synechococcus* are unknown, particularly for the form and precise location of the Si. Given this hypothesis, with this review, we discussed recent findings that small picophytoplankton may have a quantitatively significant contribution to both bSi pool and its rate of production for the global ocean. We hope this review will provide some inspirations for researchers to explore the mechanistic role of these fascinating small cells in the biogeochemical Si cycle, as well as providing a novel context of biological and ecological functions to those interested in marine Si cycle.

## The Role of Large Diatoms in Marine C and Si Cycles

Terrestrial dissolved silicic acid (DSi) through rivers and atmospheric deposition are eventually transported to the ocean. The DSi is subsequently absorbed by large diatoms to construct their cell walls, becoming an essential nutrient for diatom growth, metabolism and reproduction. Furthermore, the biological silicate, as an effective pH buffer for intracellular metabolism of diatoms, contributes to more absorption of CO_2_, which in turn encourages the large diatoms to carry out more efficient photosynthesis. Thus, large diatoms account for ~25% of the total global C sequestration, and play an important role in the regulation of atmosphere CO_2_ as well as global climate change (Field et al., 1998; Ragueneau et al., 2000; Jin et al., 2006). Due to the density-driven particle deposition, large diatoms have long been thought to be responsible for the transport of organic matter to the deep ocean (~1.5–2.8 Gton C year^−1^; Treguer et al., 1995; Armstrong et al., 2001). Altogether, large diatoms dominate the Si absorption, metabolism, conversion, and deposition in the ocean, and closely combine the Si cycle with the C cycle, which plays an important role in the start-up and continuity of the marine biological pump (Ragueneau et al., 2006).

The process by which different phytoplankton convert CO_2_ from the atmosphere into organic C through photosynthesis and send it to the deep ocean is called the “biological C pump.” In this process, Si plays an irreplaceable role in the primary production and output dominated by large diatoms. Thus, the biogeochemical cycles of C and Si are closely coupled through the growth and metabolism of large diatoms, and the “biological C pump” in the ocean is called the “biological Si pump” (Raven and Falkowski, 1999). For example, studies on long-term algal blooms and nutrient status in different nearshore areas have reported that higher N and P inputs increase the C biomass of large diatoms and simultaneously result in increased Si deposition rates (Officer and Ryther, 1980). In recent years, however, studies in some oligotrophic areas have found that the C biomass of large diatoms is relatively low, but compared with the low C biomass, the Si storage and output flux are very high. The implication is that the production and output of C and Si are obviously unbalanced in these areas, i.e., the decoupling phenomenon (Assmy et al., 2013). For example, approximately 50%–75% of the Si around Antarctica is buried in deep-sea sediments, whereas large diatoms contribute only 2%–38% of the total phytoplankton C biomass (Hedges and Keil, 1995). Similarly, a large number of sediment trap data have shown a poor correlation between the C and Si flux of large diatoms in the ocean (Klaas and Archer, 2002). Although there are other siliceous organisms (e.g., silicoflagellates and Rhizarians) in the ocean that can absorb Si, their species and abundance in the ocean are very small, their growth and sedimentation rates are very low, and their contribution to offshore silicon stock, production, and output is negligible compared to that of large diatoms. Therefore, these non-diatom siliceous groups are not the main cause of the imbalance between the production and output of C and Si. So what accounts for the imbalance between the production and output of C and Si in these oligotrophic areas?

## Advance in the Role of Picophytoplankton in Ocean Si Cycling

Up to date, it is generally believed that large diatoms transport dissolved Si from the surface to the deep ocean, dominating the global ocean Si cycle and becoming the main bridge between the oceanic C and Si cycles (Raven and Falkowski, 1999). As discussed above, it is difficult to explain the imbalance between the production and output of C and Si in the ocean (especially in oligotrophic areas) from the perspective of “only diatoms.” Interestingly, *Synechococcus* are found to accumulate substantial amounts Si, and their cells have different Si/P and Si/S ratios. Given the phylogenetic and taxonomic similarities between *Synechococcus* and *Prochlorococcus* (Kent et al., 2019), it is possible that *Prochlorococcus* also can accumulate Si, but they are too small (~0.6 μm) to detect their intracellular Si content. Regardless of the morphology of Si in these picocyanobacteria, if *Prochlorococcus* can accumulate Si, their high abundance and worldwide distribution suggest that they would have an important effect on the oceanic Si cycle. In addition, recent studies have shown that there are a large number of small diatoms genus *Minidiscus* in the picoplanktonic size-class, which may have important implications for the marine Si cycle (Leblanc et al., 2018a). Meanwhile, there is accumulating evidence that marine picophytoplankton are the important contributors of primary productivity in oligotrophic waters, and their contribution to the export of organic particles to the deep ocean could not be ignored (Richardson and Jackson, 2007). The discovery of picophytoplankton Si accumulation is likely to change the absolute role of large diatoms in the global marine Si cycle and provides us with a new perspective to link the interaction of C and Si cycles.

In some cases, the Si concentration in *Synechococcus* cells can exceed that of large diatoms, the indication being that picocyanobacteria may have a previously unrecognized and important impact on the ocean’s Si cycle (Baines et al., 2012). Thereafter, a series of studies on Si accumulation in picophytoplankton have explored the potential impact of these small cells on the ocean Si cycle. For example, Tang et al. (2014) have found that Si can be deposited on extracellular polymeric substance (EPS) associated with decomposing *Synechococcus*, which is similar to the micro-blebs observed in the deep ocean in morphology and composition. So the EPS-Si produced by the *Synechococcus* decomposition may be the precursors of the micro-blebs that may be important to Si cycling and may further enhance export of picophytoplankton to the deep ocean. Ohnemus et al. (2016) have measured the intracellular Si content of the North Atlantic, and revealed that the intracellular Si content of *Synechococcus* varied greatly, ranging from 1 to 4,700 amol Si cell^−1^. Subsequently, they have provided evidence that the variation of intracellular Si content with depth may be related to the difference of dominant clades in different water layers. Brzezinski et al. (2017) have reported that the growth rate of *Synechococcus* is not affected by the concentration of Si in culture. However, they also proposed that there are two Si pools, i.e., soluble and insoluble, in the cells of *Synechococcus*, and speculated that soluble Si would likely bind to organic ligands. Krause et al. (2017) have analyzed the Si stock and production rate of the pico-sized fraction in the Sargasso Sea, and average bSi stock and production rate accounted for 14 and 16% of the total, respectively, indicating that the picophytoplankton accounted for a large proportion of the total bSi stock and production. They also estimated the contribution of *Synechococcus* to the total pico-sized bSi standing stock (~15%) and production rate (~55%), suggesting that more than half of the Si production of picophytoplankton is likely to originate from *Synechococcus*. Ohnemus et al. (2018) have explored the chemical form of Si in the *Synechococcus* cells, and revealed that *Synechococcus* Si was spectroscopically different from the opal-A precipitated by large diatoms. Leblanc et al. (2018b) have shown that the contribution of picophytoplankton to the total bSi stocks could not be overlooked (about 11%–26% of the total bSi stocks) in the tropical South Pacific, and have also highlighted the importance of Si uptake by *Synechococcus* in the ocean.

Since Baines et al. (2012) first discovered the Si accumulation of *Synechococcus*, there have been a series of reports on the contribution of marine picophytoplankton to bSi standing stocks and production in different oligotrophic waters (e.g., the Eastern Pacific, North Atlantic, and South Pacific). Although the absorption mechanism and environmental regulation mechanism of picophytoplankton Si accumulation are not clear at present, their Si accumulation contributes significantly to the bSi standing stock (11%–50%) and production (~55%) in different water layers (Ohnemus et al., 2016; Krause et al., 2017; Wei et al., 2021a,b). Conceivably, Si accumulation by picophytoplankton may affect their growth and metabolism, and then regulate their photosynthetic C fixation and biological C pump. More importantly, these small cells may promote the sinking of particulate matter, and all organic C contained therein through their dense siliceous cells or extracellular EPS-Si into the ocean without entering the recycling process (Tang et al., 2014; Wei et al., 2022). This potential sinking process is likely to combine the Si cycle with the C cycle, making it a new bridge connecting the interactions of the oceanic C and Si cycles. Taken together, the Si accumulation of picophytoplankton not only changes our previous understanding that large diatoms mainly control the global oceanic C and Si cycles, but also plays an important role in the sequestration of C and Si in the ocean.

## Significant Contribution of Picophytoplankton to Si Budgets

Based on our previous studies in the oligotrophic open oceans, it is concluded that marine picophytoplankton could represent on average ~50% of total bSi standing stocks, which is a significant contribution (Wei et al., 2021a,b). In the Sargasso Sea, the picophytoplankton generally contributed measurable, and at times significant proportion of both the total bSi standing stocks (9%–24%) and production rates (1%–37%; Baines et al., 2012; Krause et al., 2017). Likewise, Leblanc et al. (2018b) have revealed a non-negligible contribution of the picophytoplankton to bSi stocks and production rates in the tropical South Pacific, representing 11–26 and 11–32%, respectively, of the total. As a result, these strong evidences suggest the need to evaluate the contribution of picophytoplankton to global ocean Si cycle, which may be more prominent in oligotrophic gyres where large diatoms are in low abundance. A revised global contribution of only 13 Tmol Si yr.^−1^ gross Si production in the mid-ocean gyres has been estimated by Nelson et al. (1995) and Brzezinski et al. (2011), i.e., ~5%–7% of the budget calculated for the global ocean of 240 Tmol Si year^−1^. The range in the calculation of the global bSi production rates for mid-ocean gyres is of 0.2–1.6 mmol m^−2^ day^−1^ (Nelson et al., 1995). Our previously measured production rates in the nutrient-depleted Indian Ocean for picophytoplankton (1.1–2.2 mmol m^−2^ day^−1^) may increase the contribution of the oligotrophic waters to global bSi productivity (Wei et al., 2021b). The implication is that marine picophytoplankton, which is likely to have been included in previous analyses, may contribute even more to total bSi standing stocks and production rates of the world ocean, especially in nutrient-poor waters.

However, Krause et al. (2017) have put forward a different assumption and suggested an important role for diatom-derived bSi detritus in the pico-sized fraction. In other words, the contributions of small fragments from large diatom frustules or other siliceous microphytoplankton may increase the measurable bSi standing stock of picophytoplankton, as the preponderance of the total Si pool in the ocean is detrital and not associated with living cells. The implication is that picophytoplankton have a small contribution to total bSi stocks, which may be masked by a dynamic Si pool driven by large diatoms. This different assumption appears to be invalidated, because (*i*) there is surprising similarity in morphology and composition between EPS-Si and micro-blebs (a group of marine detritus enriched in Si) and thus EPS-Si may be a precursor of micro-blebs, whereas newly produced EPS-Si may most likely originate from picocyanobacteria (Tang et al., 2014); (*ii*) Tang et al. (2014) have provided evidence that *Synechococcus*-derived bSi standing stock accounts for 50% of the bSi inventory in the surface water, thus implying that half of the bSi in the surface water may originate from *Synechococcus*; (*iii*) our earlier work has revealed that the contribution of the pico-sized fraction to total Si uptake rates is really surprising, ~44%, thus can provide insight into the significant picophytoplankton contribution to Si pool (Wei et al., 2021b); and (*iv*) some small diatoms (e.g., *Minidiscus*) are globally overlooked but play a role in the marine Si cycle (Leblanc et al., 2018a). Therefore, we suggest that the source of potential Si detritus in small size fraction may originate mostly from picophytoplankton in the ocean.

Based on our previous data in the eastern Indian Ocean (Wei et al., 2021b), we made a rough estimate for the global oceanic bSi standing stock, production rate, and export flux of picophytoplankton, although such a global estimate for these small cells had significant uncertainty. The estimated results provided a global picophytoplankton bSi standing stock of 1.55–3.85 Tmol Si based on the derived Si/C ratio (i.e., ~0.035). Leblanc et al. (2012) have previously reported a first-order estimate of the diatom C biomass for the global ocean, ranging from 444 to 582 Tg C, which converts to about 3–4 Tmol Si. Thus, the estimated global bSi stock of picophytoplankton here is similar to that of large diatoms. Furthermore, we also implied a global productivity range of picophytoplankton between 78 and 194 Tmol Si year^−1^, which is about 32%–80% of the total global annual ocean bSi production estimate (~240 Tmol Si year^−1^; Nelson et al., 1995; Brzezinski et al., 2011). Richardson and Jackson (2007) have suggested that the average C export flux of picophytoplankton in oligotrophic waters is ~6.21 mmol m^−2^ day^−1^, accounting for ~50% of the total C export flux. Converting picophytoplankton C to Si export flux, using the above derived Si:C ratio (~0.035), provides an estimated global picophytoplankton Si export flux of 0.22 mmol m^−2^ day^−1^, accounting for ~55% of the global annual ocean Si flux. Altogether, these results suggest an important role for small-sized plankton in the marine Si cycle at regional and global scales, but those calculations have large uncertainty due to the lack of data, and hence more data are necessary to better understand the spatial extent of the picophytoplankton role in the global Si cycle.

## Future Perspectives

The biological importance of picophytoplankton in the biogeochemical cycles is even more intriguing under climate change scenarios, as the result of several studies that predict shifts toward smaller species as oceans warm. Along with the discovery of Si accumulation by picocyanobacteria, their influence could be substantially larger in various aquatic ecosystems now and in future. Meanwhile, this discovery has also strong implications for the marine C and Si cycles. Hence, this review highlights the significant advances that have been made in the past decade toward improving our understanding of Si accumulated by picophytoplankton, and their ecological and biogeochemical impacts on the Si cycle. Although this review fills some identified knowledge gaps, there are three main aspects that still need to be addressed in the future: (*i*) our understanding of the factors controlling the magnitude and variability in the pico-sized contribution to both total bSi stocks and its rate of production is not enough; (*ii*) the physiological and/or biological mechanisms of Si accumulation by picocyanobacteria are not clear; and (*iii*) the picophytoplankton Si export associated with possible pathways has not been established.

## Author Contributions

JS conceived the original idea and defined the manuscript content. YW wrote the paper. All authors contributed to the article and approved the submitted version.

## Funding

This work was financially supported by the Project funded by China Postdoctoral Science Foundation (2021M703590), the Shandong Postdoctoral Innovation Talent Support Program (SDBX2021014), the National Nature Science Foundation of China grants (41876134), the Central Public-interest Scientific Institution Basal Research Fund, YSFRI, CAFS (20603022022010), the Qingdao Postdoctoral Applied Research Project, and the State Key Laboratory of Biogeology and Environmental Geology, China University of Geosciences (GKZ21Y645).

## Conflict of Interest

The authors declare that the research was conducted in the absence of any commercial or financial relationships that could be construed as a potential conflict of interest.

## Publisher’s Note

All claims expressed in this article are solely those of the authors and do not necessarily represent those of their affiliated organizations, or those of the publisher, the editors and the reviewers. Any product that may be evaluated in this article, or claim that may be made by its manufacturer, is not guaranteed or endorsed by the publisher.
